# Exploring how Australian general practice registrars define cultural safety with Aboriginal and Torres Strait Islander patients: a mixed method study

**DOI:** 10.1186/s12875-024-02422-4

**Published:** 2024-05-16

**Authors:** Kay Brumpton, Hannah Woodall, Rebecca Evans, Henry Neill, Tarun Sen Gupta, Lawrie McArthur, Raelene Ward

**Affiliations:** 1 Griffith University, Gold Coast Campus, Southport, Australia; 2 Rural Medical Education Australia, 190 Hume Street, East Toowoomba, QLD 4350 Australia; 3 James Cook University, Townsville, Australia; 4 The University of Adelaide, Adelaide, Australia; 5 University of Southern Queensland, Toowoomba, Australia

**Keywords:** General practice, Registrars, Indigenous, Cultural safety, Definition

## Abstract

**Background:**

Understanding how the general practice medical workforce defines cultural safety may help tailor education and training to better enable community-determined culturally safe practice. This project seeks to explore how Australian general practice registrars define cultural safety with Aboriginal and Torres Strait Islander patients and alignment with an Australian community derived definition of cultural safety.

**Methods:**

This mixed method study involved a survey considering demographic details of general practice registrars, questionnaire, and semi-structured interviews to explore how general practice registrars defined cultural safety and a culturally safe consultation.

**Results:**

Twenty-six registrars completed the survey. Sixteen registrars completed both the survey and the interview.

**Conclusion:**

This study shows amongst this small sample that there is limited alignment of general practice registrars’ definitions of cultural safety with a community derived definition of cultural safety. The most frequently cited aspects of cultural safety included accessible healthcare, appropriate attitude, and awareness of differences.

## Background

In the context of Australian general practice, understanding how the general practice medical workforce defines cultural safety for Australia’s Indigenous Aboriginal and Torres Strait Islander people is important. The practice of culturally safe general practice is an Australian national health priority. The Australian National Scheme’s Aboriginal and Torres Strait Islander Health and Cultural Safety Strategy 2020–2025 vision statement is that patient safety is the norm forAboriginal and Torres Strait Islander Australians [1].

Australia uses an apprenticeship model of work-based, experiential learning for medical graduates training to be specialist General Practitioners (GPs) [2]. These doctors, referred to as GP registrars, have completed medical school and at least mandatory hospital training time. During this training registrars are provided with cultural safety (or similar) training and are expected to demonstrate effective and culturally competent communication and care for Aboriginal and Torres Strait Islander Australians [3, 4]. This is supported by the Aboriginal and Torres Strait Islander Health Curriculum Framework that describes graduate learning outcomes including culturally safe communication [5].

Historically within Australia there has been no consistent definition for cultural safety [6–8]. Cultural safety was introduced as a concept in 1992 by Māori nurse Ramsden [9]. Since then the medical education literature has used many terms for this and similar concepts [6]. For example, the Aboriginal and Torres Strait Islander Health Curriculum Framework uses cultural awareness, safety, competence, capability, responsiveness, security and respect [10]. Others use appropriate [11], humility [12] and desire [13]. Educationalists such as Ryder [14] see awareness, sensitivity, and safety as a progression of skill. The origin of these definitions and the cultural voice in determining these definitions is frequently absent or unclear [15]. However, in December 2019, the Australian Health Practitioner Agency (AHPRA) released a consensus statement defining cultural safety [16]. This consensus statement was arrived at through a consultation and consensus process led by the Aboriginal and Torres Strait Islander members of the AHPRA Aboriginal and Torres Strait Islander Health and Cultural Safety Group [16] (Table 1).

**Table 1:** AHPRA consensus statement of cultural safety [1, 16]

AHPRA consensus statement of cultural safety: “*Cultural safety is determined by Aboriginal and Torres Strait Islander individuals, families and communities. Culturally safe practise [sic] is ongoing critical reflection of health practitioner knowledge, skills, attitudes, practicing [sic] behaviors and power differentials in delivering safe, accessible and responsive healthcare free of racism.*”
AHPRA further states how an individual health practitioner demonstrates culturally safe clinical practice. Specifically, this requires the individual to: *- “Acknowledge colonisation and systemic racism, social, cultural, behavioural and economic factors which impact individual and community health* *- Acknowledge and address individual racism, their own biases, assumptions, stereotypes and prejudices and provide care that is holistic, free of bias and racism* *- Recognise the importance of self-determined decision-making, partnership and collaboration in healthcare which is driven by the individual, family and community* *- Foster a safe working environment through leadership to support the rights and dignity of Aboriginal and Torres Strait Islander people and colleagues.”*

As identified by the Cultural Safety Strategy 2020–2025, a key element of achieving cultural safety is ensuring a consistent definition of cultural safety [1]. The AHPRA definition provides a baseline reference for defining cultural safety in Australia and as such, we have used this definition for this research. GP registrars, who have currency of clinical practice and are actively studying for their specialty GP examinations, were considered on principle, the most likely within the GP medical workforce to be aware of the AHPRA definition of cultural safety. If this cohort of GPs do not have a common understanding of cultural safety, or a working knowledge of the AHPRA definition, it would suggest there is opportunity for Australian GP training to adapt teaching to address this gap. Furthermore, there is considerable advantage in understanding this issue early in one’s career, with many years of potential benefit for patients and communities; it also may point to an important curriculum group: not just what do we teach and when, but how do we teach it so registrars internalise the message and can incorporate this into their practice.

Understanding how the GP medical workforce defines and views cultural safety may help tailor education and training to better enable community-determined culturally safe practice. This project seeks to explore how GP registrars define cultural safety and alignment with the AHPRA definition of cultural safety.

## Methods

### Research design

A detailed description of the methods for this research has been published [17]. A pragmatic approach was used to allow the Indigenous researchers’ expertise and perspectives to be privileged without conforming to a western framework. This phase of the study involves a mixed method approach to understand how GP registrars define, develop, and demonstrate cultural safety with Aboriginal and Torres Strait Islander patients. This paper reports on one component of the research: how GP registrars define cultural safety.

The [deidentified for review (DFR)] Human Research Ethics Committee approved this study (H8296) following review by Aboriginal and Torres Strait Ethics Advisors in accordance with the National Health and Medical Research Council guidelines [18]. Indigenous governance for this project is undertaken by a community reference group who are associated with an Aboriginal community-controlled health organisation and recognised the need for this study. Aboriginal and Torres Strait Islander participants were not purposively recruited for this phase of the research project.

### Participants

GP registrars were chosen as a purposive sample of the GP medical workforce as they were more likely to have participated in recent cultural safety education as part of their training. All GP registrars undertaking active training with an Australian regional GP registrar training organisation (RTO) were invited to participate in the study. Registrars with this RTO work across a diverse range of Indigenous and non-Indigenous communities. Informed consent was obtained from all subjects.

### Data collection

Data collection techniques was in two parts and was conducted from March to August in 2022, more than two years after the release of the AHPRA consensus statement. Part 1 involved administering a Qualtrics based survey considering demographic details of the GP registrars and select questions from West’s cultural capability measurement tool (CCMT) [19], Ryder’s measurement of attitude change (MAC) [14]) and the Self-Reflection and Insight Scale (SRIS) [20]. As queries in the CCT and MAC overlap we preferentially chose questions from the MAC as it had previously been used with medical students, whereas the CCMT was mostly nursing focussed. A table comparing and presenting the chosen queries, and adapted wording, is available in the previously published methods [17]. In this paper we report on the following survey questions that were grouped, by A7 an Indigenous research academic and A1, against the AHPRA definition components of ongoing critical reflection, knowledge, and attitude (Table 2).

**Table 2:** Survey questions

Item^a^	Item query
On going critical reflection	
MAC	I think my beliefs and attitudes are influenced by my culture
MAC	A GPs’ own cultural beliefs influence their health care decisions
MAC	Time in the GP curriculum devoted to the promotion of^a^ self-awareness and well-being is time well spent
CCMT	I find it difficult to understand the beliefs of different cultural groups
SRIS	I do not often think about my thoughts
SRIS	I am not really interested in analyzing my behavior
SRIS	It is important for me to evaluate the things that I do
SRIS	I am very interested in examining what I think about
SRIS	I do not really think about why I behave in the way that I do
Knowledge	
MAC	All Australians need to understand Aboriginal and Torres Strait Islander history and culture
CCMT	History does not impact on Aboriginal and Torres Strait Islander health
CCMT	Understanding Aboriginal and Torres Strait Islander peoples’ history will inform my practice as a GP
CCMT	Understanding Aboriginal and Torres Strait Islander peoples’ social practices will not apply to my practice
Attitude	
MAC	Aboriginal and Torres Strait Islander people, due to the own cultural beliefs and values, have the poorest health status in Australia
MAC	Aboriginal and Torres Strait Islander people should be responsible for improving their own health
MAC	Aboriginal and Torres Strait Islander people should not have to change their culture just to fit in
MAC	I have a social responsibility to work for changes in Aboriginal and Torres Strait Islander health
CCMT	Aboriginal and Torres Strait Islander peoples receive unnecessary special treatment from government

*MAC* Measure of attitude change [14], *CCMT* Cultural capability measure [19], *SRIS* Self-reflection and insight scale [21]

^a^Survey item origin

Part 2 involved semi-structured interviews with GP registrars to explore how they define cultural safety and if they describe the AHPRA definition, or components of this definition [17]. The questions analysed for this paper included:

1. Could you please define for me the concept of cultural safety?
2. Why do you think Aboriginal and Torres Strait Islander patients choose (would choose) to see you as their doctor?
3. What are the most important things you do (could do) to make Aboriginal and/or Torres Strait Islander patients feel culturally safe when you are consulting?
4. Can you describe a time when you feel a patient may have felt culturally unsafe when you were consulting?
5. What does a culturally safe GP consultation look like to you?

### Data analysis

Survey data was descriptively analysed to both characterise the cases and provide contextual data for assisting in interpreting the interview data. One Aboriginal and Torres Strait Islander member of the research team was a novice researcher. Data was presented using a dynamic framework to allow them to make sense of the stories collected. Transcripts were initially studied using a content conceptual analysis approach to reduce the text to manageable content categories [22]. Components of the AHPRA consensus statement were used as initial categories with flexibility to add additional concepts. The AHPRA consensus statement does not define the terms used within the definition. As such, some quotes were classified to more than one category to capture this nuance rather than create artificial division. Collaborative research yarning, a conversation facilitated by the Aboriginal and Torres Strait Islander members of the research team to discuss concepts and ideas, was used to decide on categorisation and to identify key themes [23]. NVivo® analysis software and Excel were used when coding data, recording frequency of occurrence of items of interest, and collating key concepts. The survey results and interview data are reported together to build a richer understanding of how registrars view and define cultural safety.

### Reflexivity

The principal investigator Author (A) 1 is an experienced GP academic working in an Aboriginal Medical Service. A2 a GP researcher, A3 a senior researcher, A4 an Aboriginal cultural educator for the RTO, A5 and A6 are clinical academics, and the latter was director of the RTO who had overall responsibility for registrar training. A7 is an Aboriginal academic from Kunja Nations. The research assistant is an evaluation coordinator with the RTO and conducted recruitment and registrar interviews. A6 had no role in recruitment and had access to only de-identified data. A community advisory group of Aboriginal and Torres Strait Islander people have been involved in the research since inception and worked with A1 and A3 to design the project and yarned about the data and findings. All researchers participated in all other phases of the project.

## Results

### Participant characteristics

A total of 26 registrars responded to the recruitment email and completed the survey. Of these, 16 registrars arranged interviews upon first contact. As there were no new insights or information arising from the collected data after these 16 interviews, we did not schedule further interviews. The survey data for both groups of registrars (survey only or survey and interview) were similar (Table 3). Most registrars were less than 34 years old, had graduated from an Australian university in the last seven years and had limited experience in Aboriginal and Torres Strait Islander health. Registrars from the two Australian GP training colleges (Royal Australian College of General Practitioners and Australian College of Rural and Remote Medicine) were equally represented. All registrars had participated in cultural education training. Two registrars self-identified as Aboriginal and Torres Strait Islander.

**Table 3:** Characteristics of participating registrars

Registrar characteristic		Registrars who participated in survey and interviews *N* = 16	Registrars who participated in survey only *N* = 10	Total registrars *N* = 26
**Age**	25–34 years	10	6	16
	35–44 years	5	3	8
	> 44 years	1	1	2
**Gender**	Female	11	8	19
	Male	5	2	7
**Post-graduate year**	1–4 years	6	4	10
	5–7 years	8	3	11
	8–10 years	1	2	3
	11–15 years	1	1	2
**Experience in Aboriginal and Torres Strait Islander health**	Nil	8	4	12
	< 1 year	4	4	8
	1–3 years	3	1	4
	> 3 years	1	1	1
**Medical degree**	Preferred not to state	1	0	1
	An Australian University in Queensland (the state where the research was conducted)	11	5	16
	Other Australian university	4	2	6
	International university	0	3	3
**Time lived in Australia**	6–10 years	5	2	7
	11–15 years	1	1	2
	> 15 years but not all their life	2	1	3
	All their life	8 (including 2 Aboriginal and Torres Strait Islander registrars)	6	14

A total of 618 minutes of audio-recording was analysed. The median length of interviews was 33.4 minutes with the longest interview 95 minutes and the shortest 18 minutes.

We describe how registrars define and describe cultural safety using the AHPRA definition as a framework, artificially separated into its individual components. The survey and interview findings are reported together under each category.

## Determined by Aboriginal and Torres Strait Islander people

When asked to define cultural safety, no registrars explicitly indicated that cultural safety should be determined by Aboriginal and Torres Strait Islander Australians. No registrars referred to the AHPRA consensus statement of cultural safety. Two registrars alluded to Aboriginal and Torres Strait Islander health professionals determining and delivering culturally safe care.

You don't know what you're doing wrong [in culturally unsafe consultations]. But I can ask...some Indigenous colleagues, like, was that good? (6389)

I don’t think we actually have any Indigenous or Torres Strait Islander doctors at the moment, but it would be nice if there was someone and I could say, “Listen, if you’re not comfortable speaking to me, I have my colleague – she’s wonderful, she’s also Indigenous, if you prefer to speak to her”. (3270)

An Aboriginal and Torres Strait Islander registrar discussed the challenges for Indigenous (and perhaps non-Indigenous) health practitioners managing patients’ expectations when cultural safety is determined by Aboriginal and Torres Strait Islander peoples and there is a resultant agenda mismatch between patient and clinician.

Seeing, working in smaller regional areas where it is well known that there's an Aboriginal doctor there, sometimes there's expectations from Indigenous patients that you will help me with X, Y and Z, and if you don't, you're not helping Aboriginal people as a whole. I've definitely had situations where Indigenous patients have said, “You know, your mother would be ashamed of you”, or “You're acting white” because I don't do X, Y and Z. Where they would never have said that to a non-Indigenous doctor.” (8230)

### Ongoing critical reflection

Several registrars recognised the importance of reflective practice and addressing bias. They discussed the negative impact of colonisation on patients and of having insight into their own culture and acceptance of alternative beliefs.

[Cultural safety teaching] makes people really reflect on the history of Australia, what other people's experiences are. Brings that to the sort of forefront of their mind that this could be something that this particular person in front of me is experiencing. And how can I make this transaction more culturally safe for this person?” (7400)

…being respectful of traditions and kind of being accepting of the differences between maybe your personal culture and what your patients’ culture might be and just being very respectful of that difference. (6278)

Three of the registrars interviewed were unable to identify any consultations that were culturally unsafe. Other registrars, when describing culturally unsafe encounters, detailed that they may be unable to recognise culturally unsafe care.

I'd like to think I'd be able to recognize it [culturally unsafe care], but I wonder sometimes if it happens without us really knowing. (7400)

**Fig. 1:**
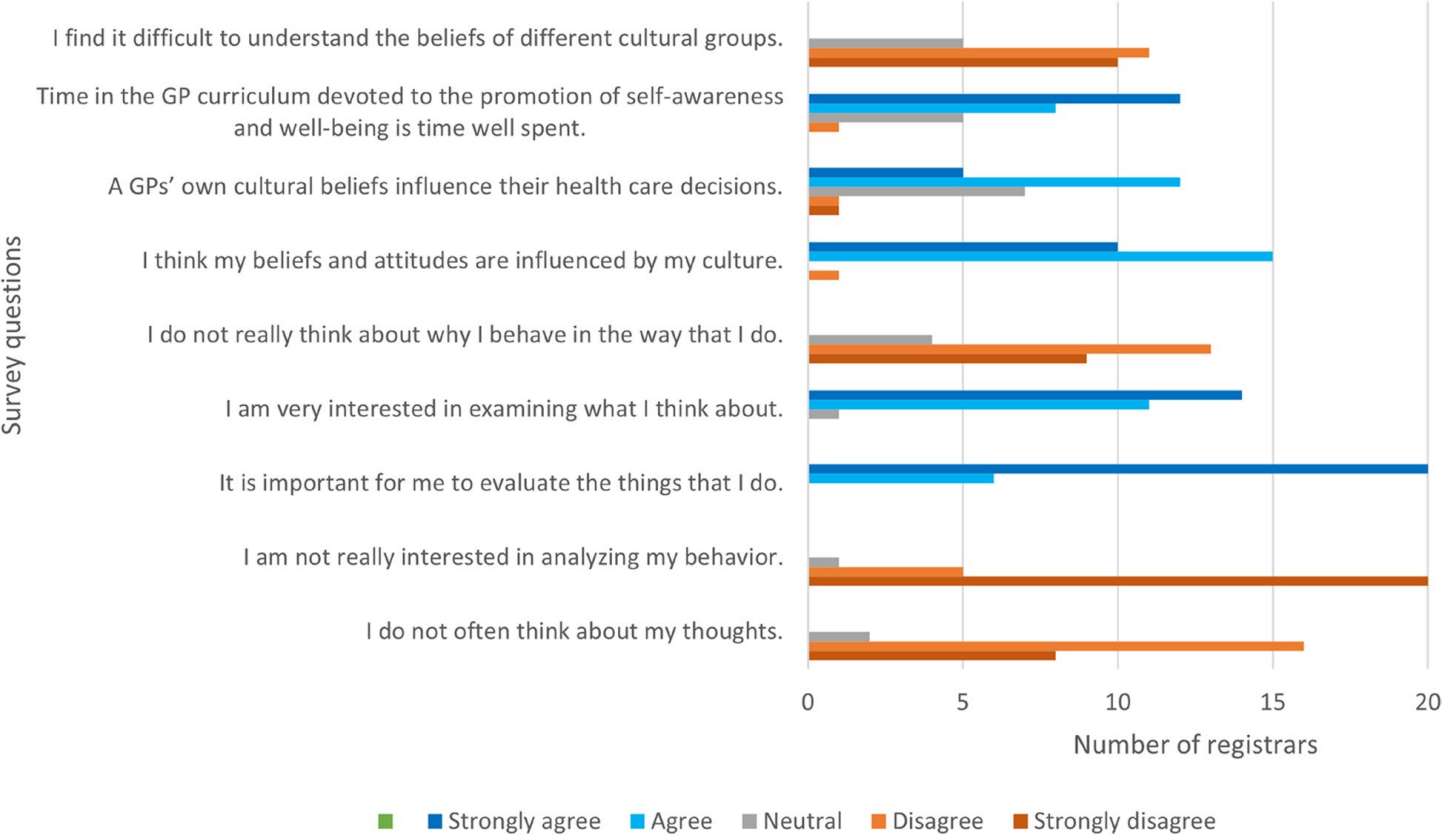
GP registrar self-reported response to critical reflection on cultural safety

In the survey, most registrars reported reflective practice, the importance of self-reflection, and that their beliefs and attitudes are influenced by their culture (Fig. 1). Some registrars indicated they were not particularly interested in self-reflection but recognised the importance of this. For example, one registrar indicated they were neutral in terms of interest in analysing their behaviours but strongly agreed that it is important to evaluate the things that they do (Fig. 1). Most registrars saw the development of self-awareness in the GP training curriculum as valuable (Fig. 1). In most survey questions there are a small number of outliers. These outlying responses are from a variety of registrars, and not the same registrar with extreme views.

### Knowledge

**Fig. 2:**
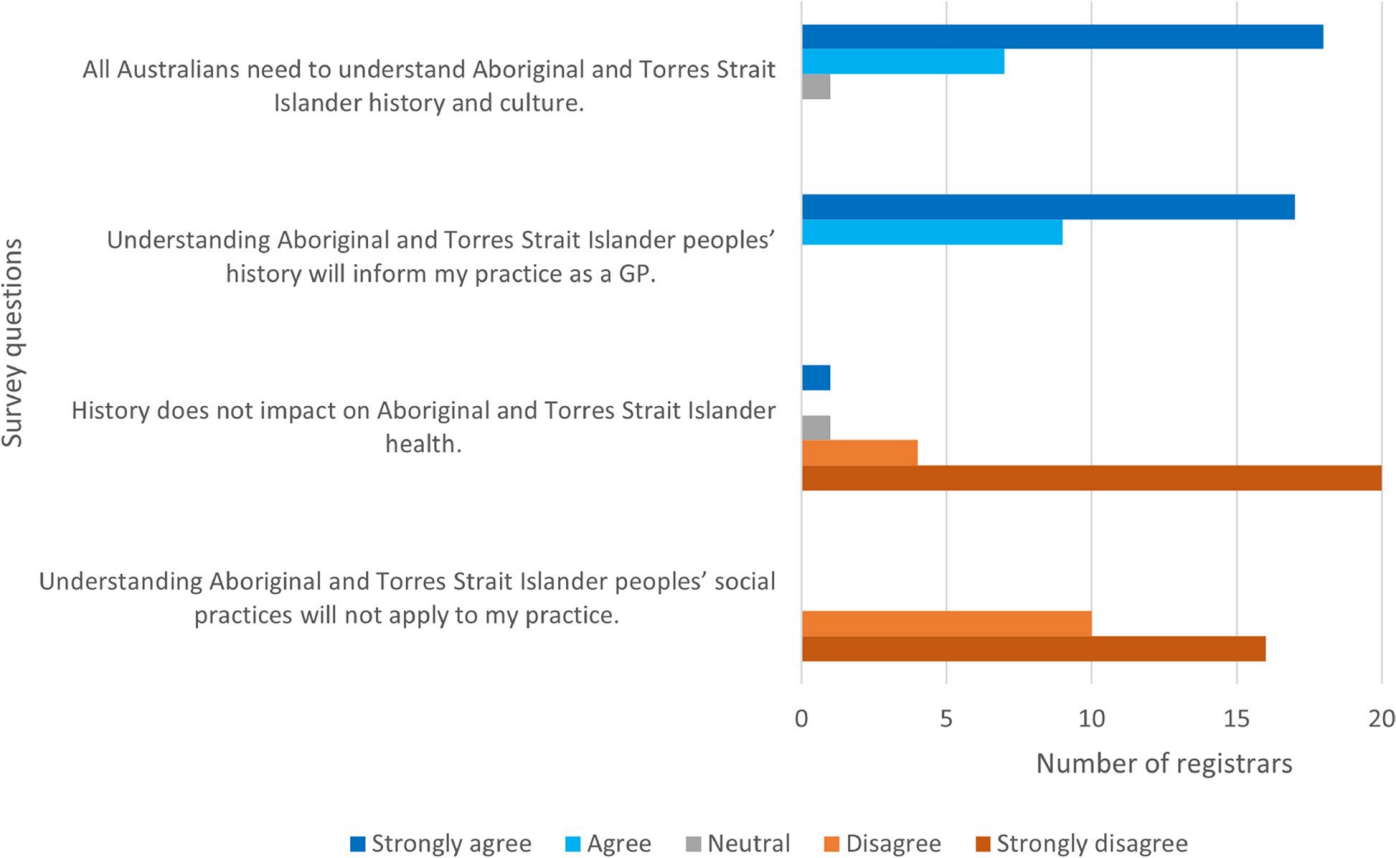
GP registrar views on importance of history and social practices when consulting with Aboriginal and Torres Strait Islander patients

During the interviews, opinion on the importance of knowing about Australian history pre-colonisation was polarised and either considered important or not important to know. However, all registrars considered it very to extremely important to know about Aboriginal and Torres Strait Islander history post-colonisation. In the survey all registrars considered that an understanding of history will inform clinical practice (Fig. 2). Only one registrar described the importance of knowing the history of Aboriginal and Torres Strait Islander people to define culturally safe care:

So, practising cultural safety includes an understanding of history and what has gone on before, especially in our own industry…especially given for a lot of our First Nations patients as that intergenerational trauma that comes from all kinds of things. (4091)

In contrast to this, two registrars indicated that history does not impact on Aboriginal and Torres Strait Islander health. Four registrars described in the interview that they had very little knowledge of Australian history. These four registrars all completed medical school in Australia and had completed cultural competency (or similar) training. One of these registrars had lived in Australia all their life.

Several registrars expressed surprise at learning of the recency of historical events that have impacted on the health and wellbeing of Aboriginal and Torres Strait Islander peoples.

...take the time to learn the history and how recent it was. There is a lot of unfair judgment and bias and I think it comes because they don't actually know what's happened. And then learn it and put yourself in those shoes. That's probably the first thing I would say. Imagine you, your family and everybody in that situation and then see if you would still feel the same way about someone who acted in a certain way that you were you were about to judge. (2601)

An Aboriginal and Torres Strait Islander registrar encouraged medical students and registrars to learn “the true” history of Australia and …”*to fathom the risk factors and how the impact of colonisation has had*” (8230) for Aboriginal and Torres Strait Islander patients.

### Skills and practicing behaviours

When asked to define cultural safety most registrars (*n* = 10) described cultural safety as being aware of and respecting cultural beliefs and customs.

I would say that it's just being aware of someone's cultural background and how that might affect, I guess, the whole trajectory and interaction with that patient, with that person. You know, it's extending from their social circumstances, their interpretation of just language. (6389)

…acknowledging that that many people have an aspect of their culture or their heritage that impacts their way of life and their way of thinking and their way of approaching things and understanding that due to that, people can approach things very differently, especially in the medical field. They have a custom or a way that they deal with things and understanding that and respecting that, but also acknowledging that, “Oh, this is probably very different to the evidence-based medicine I've been taught”. (3270)

Similarly, when comparing cultural safety with culturally unsafe care, registrars most frequently detailed non-adherence to local custom or assumed local custom. Registrars were most acutely aware of gender differences between them and their patients.

I had one patient that I felt I found difficult. He was an Aboriginal elder… and obviously had a lot of, standing in this community. And here I was as a young white female telling him that his erectile dysfunction can't be fixed because it was due to diabetes and chronic vascular disease. And that was I found that a very tricky consultation because he just wouldn't take my word for that... But yes, it's telling to having that discussion at the first consult with a big age gap, cultural gap, social standing gap I thought that was very challenging and I'm not sure whether he felt culturally safe or not. (7216)

Other registrars defined cultural safety as rapport building, adequate consultation time and patient follow-up. Registrars also considered patient-centred care as important when defining cultural safety.

[Cultural safety is] where the patient can be comfortable in the GP practice. They feel comfortable, will bring up any issues that they have and feel that they'll be heard, that they will be engaged and participating in whatever diagnosis, investigation, management plan that's happening and that the context of their life and their cultural beliefs, beliefs and their circumstances they involved in incorporated into that management plan. And so, you're not basically telling them that they need to go for a scan somewhere when they don't have a car. (7400)

One registrar described features of cultural safety (the patient being comfortable, respected, and able to engage in the consultation) but implied this was primarily the responsibility of the patient and not for the GP. For example, the registrar statement: “They [the patient] need to understand that they are being respected”, suggests a paternalistic attitude to the consultation process.

When both the GP and the patient, they are at ease, they are opening up, they are telling you the truth, they are not holding anything back and they are willing to show interest in what you are prescribing to them or advising to them, and they are willing to understand that it is in their best interest...So they need to understand that they are being respected and only that time they will feel that they are culturally safe environment. They are getting that culturally safe environment, and they need to understand that they are being understood and that their language is being understood and that they are their concerns are being taken seriously, and that consult then becomes a culturally safe consult. (1111)

### Attitude

Registrars described a friendly approach, being tolerant, respectful, open-minded, non-judgemental, and willing to learn.

Just be willing to have an open mind as willing to learn. I think that's the biggest thing...I don't think patients expect us to be culturally aware of everything and every possible culture. But I think as long as we're willing to learn, I think patients appreciate that. And I think if you're being honest with patients like please feel free to correct me if I'm wrong about your culture or if you feel like I'm saying anything offensive. I would be happy to correct my words, and I think people would appreciate that. If you're just being honest, if you don't know, then you don't know. (6278)

I think a culturally safe GP consultation would be for the patient to come in, not have any barriers to them accessing the health service, which sometimes there's a lot of barriers that aren't recognised to be able to freely come in and not have that, that feel of judgment, that feel of they're looking down on me or I'm here because I don't want to ask something because I don't want to sound stupid. (8230)

**Fig. 3:**
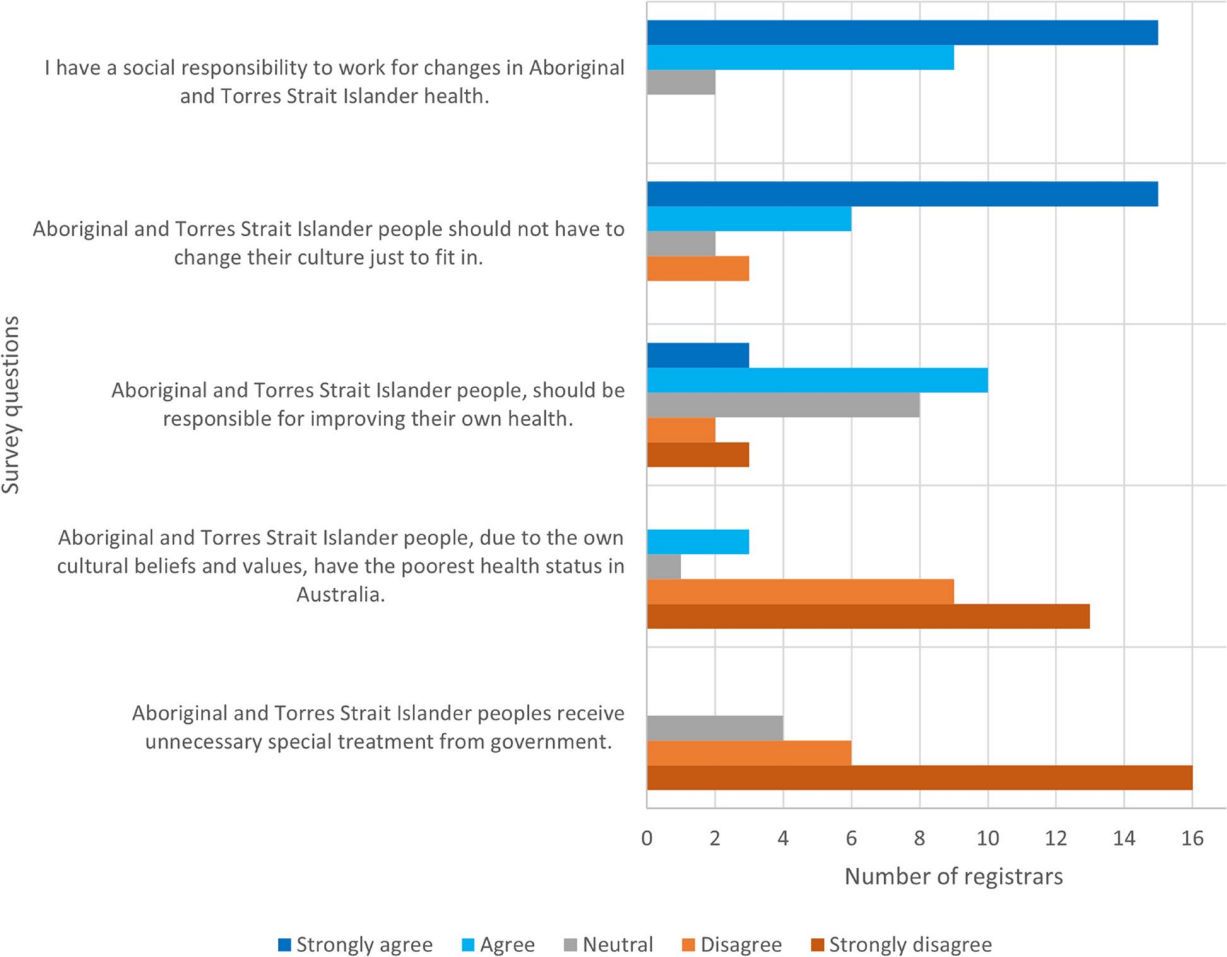
GP registrars self-reported attitude to Aboriginal and Torres Strait Islander health

Most registrars considered they had a social responsibility to work for changes in Aboriginal and Torres Strait Islander health (Fig. 3). Three registrars considered that Aboriginal and Torres Strait Islander people, due to their own cultural beliefs and values, have the poorest health status in Australia. Four registrars were neutral in their opinion on Aboriginal and Torres Strait Islander people receiving unnecessary special treatment from the government.

### Power differentials

The inherent power imbalance between a doctor and a patient was not particularly highlighted by registrars. Registrars described feeling “over-powered” by patients as demonstrated in previous quotes such as “You know, your mother would be ashamed of you”, “You're acting white”, “A big age gap, cultural gap, social standing gap”. One of the Aboriginal and Torres Strait Islander registrars described power differential in a consultation.

I have been a part of a team within a hospital environment, I have been on ward rounds where there is an Aboriginal patient in the bed and they're sitting down and there's five strangers standing around them all chatting over top of them and sort of mumble and go and they're not really sure what's happening … And I found in those situations the doctor's own beliefs and values were very much pushed on patients. (8230)

### Safe, accessible, and responsive healthcare

Registrars referred to providing a safe environment where patients feel comfortable and welcome. They provided examples of a familiar physical environment and access to Aboriginal and Torres Strait Islander staff and health professionals.

And then every step of the way, I think in a GP consultation to be culturally safe, incorporating when the patient does want, patient, other allied health professionals or Aboriginal health workers, if they need me to help them along the way, particularly if they're really new to any accessing health care, previously really bad experiences, traumatized, and that's really common as well. (7400)

…providing them [Aboriginal and Torres Strait Islander people] with an environment which is safe for them. There is no challenge to them. They don’t feel belittled, or they don’t feel any denial of who they are. So, their identity is preserved in every consult that we have with them...irrespective of their age or gender. (1111)

The two Aboriginal and Torres Strait Islander registrars defined cultural safety as having confidence to access healthcare with the absence of discrimination and judgement.

To be able to freely come in and not have that, that feel of judgment, that feel of they’re looking down on me or I’m here because I don’t want to ask something because I don’t want to sound stupid. (8230)

For me, cultural safety is somebody feeling as though they can be who they are, that they don’t have to have their guard up when they come into to see a GP because they are from Aboriginal or Torres Strait Islander background or any other group, that they feel comfortable coming to have questions around their health addressed no matter how silly that they may think that that could be or insignificant because a lot of the time getting things addressed early is actually the key to preventative health care and preventing complicated illness. So that’s what is underpinning cultural safety. If you are confident in approaching your health care provider, then that is and it’s a safe place for you to raise anything. That’s what that means for me. (9304)

### Free of racism

No registrars used the word racism when defining cultural safety. One registrar described having a consultation free of discrimination. An Aboriginal and Torres Strait Islander registrar defined cultural safety as:

Well, cultural safety for me is entering into any environment where there’s no threats or barriers to your personal well-being. So, to feel culturally safe within an environment is not to be excluded by your ethnicity in any way. (8320)

When reflecting on culturally unsafe experiences, several registrars preferentially chose alternative reasons for patient distress, such as mental illness, alcohol, or drug intoxication.

One [culturally unsafe consultation] …intoxicated patient came in and I tried to assess them. They were yelling and screaming and didn’t want intervention…But I wouldn’t put that down to culture or being Aboriginal, probably more the alcohol and being aggressive. (2797)

Some registrars recognised racism in colleagues. Another registrar described the challenge of closing the gap without being racist.

I remember I had a young woman, she was Indigenous, and she came in with fevers… my consultant who just said, “Maybe get another set of blood cultures, you know, because she is Indigenous” ….it’s almost like saying there’s something extra that we have to do because of your race. (3270)

## Discussion

This is the first known study that uses the AHPRA definition when exploring delivery of culturally safe care by GP registrars. In this study, registrars did not have a common understanding of cultural safety and no registrars referred to the AHPRA consensus statement of cultural safety. Limited alignment of participants’ definition of cultural safety with the AHPRA definition suggests that there are variations in understanding and interpretation, potentially hindering effective implementation and outcomes of cultural safety practices. Without agreement on a definition for cultural safety, registrars may continue to interpret cultural safety uniquely. The AHPRA statement was released at the end of 2019 [16]: prior to most of the registrars in this study commencing GP training. The Royal Australia College of General Practitioners (RACGP) has incorporated this definition into the curriculum since 2022 [24] and the Australian College of Rural and Remote Medicine (ACRRM) into the rural generalist curriculum since 2021 [25]. Registrars from both colleges were equally represented in this study.

Overall, registrars did not consider cultural safety being determined by Aboriginal and Torres Strait Islander patients. Cultural safety can only be determined by those who are receiving care and will be unique to everyone [26]. When the person in a position of authority or power, that is the registrar, determines culturally safe care this likely serves to increase the colonisation-based power imbalance and perpetuate racism. Without a foundational definition of culturally safe care, registrars had limited capacity to identify culturally unsafe care. Some registrars recognised they may be blind to culturally unsafe care but did not take the next step to place ownership of defining culturally safe care to Aboriginal and Torres Strait Islander peoples. There was an expectation that Aboriginal and Torres Strait Islander doctors bear responsibility for correcting culturally unsafe care, acting as a resource, and be offered to patients as a culturally safe alternative. Whilst this action does place determination of cultural safety in the hands of Aboriginal and Torres Strait Islander people, this is unsustainable and individual doctors should not have to be responsible for a culturally safe health service. This “cultural load” or “identity strain” on Indigenous medical staff is experienced similarly across other professions and workplaces [27, 28]. However, the challenge then lies in determining who is responsible for educating, mentoring, and correcting registrars to ensure delivery of culturally safe practice. Furthermore, we need to better understand how we develop cultural safety in registrars without subjecting patients to racist, culturally unsafe consultations in the process.

Registrars in this study volunteered with a willingness and want to improve health outcomes for Aboriginal and Torres Strait Islander peoples. The semi-structured interview questions encouraged critical reflection of practice. Critical reflection has been considered a crucial component of cultural safety [16]: not just what care is provided but how care is provided. The AHPRA definition calls on registrars to reflect on their knowledge, skills, attitudes, practising behaviours and respond to power differentials [16]. The problem is, most models of reflective practice ask the registrar “How do you feel about the clinical encounter?” rather than “How did the patient feel?” [29]. Borrowing from the field of psychology, Walker’s critical reflection framework of analysis and Dudgeon et al. Indigenous Community Management and Development program provides an alternative approach to critical reflection that includes questioning, analysing, defining the issue, seeking other perspectives, mapping, critical reflection through dialogue and recording activities/observations [30].

Most cultural safety (or similar) training has a focus on understanding the impact of colonisation on Aboriginal and Torres Strait Islander people [31]. Yet only one registrar discussed this when defining cultural safety. When registrars were defining cultural safety, most referred to respecting local cultural customs particularly with respect to men’s and women’s business. With most registrars primarily consulting non-Indigenous patients, awareness of Aboriginal and Torres Strait Islander cultural and social norms is important. However, this creates a risk of ‘othering’ (us versus them phenomenon) where stereotyping and discrimination occurs.

Registrars were not particularly aware of power differentials and would attempt to equalise the relationship through points of commonality. Registrars viewed safe and accessible care through the lens of a welcoming physical environment, provision of family consultations and flexible appointment times. Registrars were comfortable attributing patient distress to factors other than culturally unsafe care. There was limited recognition of how a registrar’s own beliefs and biases might influence the health care interaction.

When registrars continue to hold culturally unsafe opinions, such as it is the patient’s responsibility to respect the doctor and understand they are acting in their best interest, power imbalance and health inequity will persist. The AHPRA definition refers to ongoing critical reflection of attitude without elaborating further on what is an appropriate attitude for provision of culturally safe care to Aboriginal and Torres Strait Islander people. Registrars reported cultural safety as having a non-judgemental, respectful, open-minded approach. This aligns with Aboriginal and Torres Strait Islander perspectives on desired personality attributes of medical graduates [11].

Understanding why the AHPRA definition has not been adopted by these registrars is important. Registrar training does not occur in a vacuum and there are multiple opportunities for registrars to be onboarded to accepting and practising the AHPRA consensus statement of cultural safety. Hospital and GP supervisors, GP training organisations, medical educators, GP colleges, and cultural educators can all influence registrars’ practice. Previous research has suggested that GP Supervisors may not consider teaching of cultural safety (or similar) as a priority and formal training may assist in developing a deeper understanding of cultural safety (or similar) and standardise training [32].

### Strengths and limitations

This mixed methods study enabled integration of survey and interview data to explore how GP registrars view and define cultural safety. The participants were from differing backgrounds and had varying medical education and clinical experience. The participants were in one Australian state that is characterised by a diverse Aboriginal and Torres Strait Islander population. The sample of GP registrars was small; however, the in-depth qualitative data collected provides good insight to this sample’s perceptions of cultural safety. The data was also based on self-assessment. The consensus of practicing GPs and their understanding of cultural safety may be quite different and is worthy of further research. Further, there is possible participation bias wherein those with an interest in cultural safety may be more likely to participate. Additional response bias in the form of acquiescence bias may be present. However, the in-depth qualitative interviewing may act to mitigate some of this.

## Conclusion

This study shows amongst this small sample that there is limited alignment of GP registrars’ definitions of cultural safety with that proposed by AHPRA. The most frequently cited aspects of cultural safety by registrars included accessible healthcare, appropriate attitude, and awareness of differences. Cultural safety training may benefit from greater attention to awareness of embedded racism, power differentials, ongoing critical reflection, and enabling of self-determination of Aboriginal and Torres Strait Islander people. The alignment of an individual doctor’s understanding of cultural safety to the national AHPRA definition is vital to improve the health outcomes of Aboriginal and Torres Strait Islander people. The combined effects of colonisation, racism, marginalisation, and other social determinants of health continue to affect the health outcomes of Aboriginal and Torres Strait Islander people. This study suggests these factors, or the importance of these factors, is remaining either invisible to registrars or dismissed. Given the inclusion of cultural safety training across all sectors of health education, it is important for us to understand why this is occurring and explore ways of awakening awareness of racism in registrars’ consciousness.

## Data Availability

The datasets analysed during the current study are not publicly available due to participants being potentially identifiable from the small dataset but are available from the corresponding author on reasonable request.
